# CDK4/6 inhibitor in combination with aromatase inhibitor effective in metastatic hidradenocarcinoma

**DOI:** 10.1111/ddg.15723

**Published:** 2025-04-25

**Authors:** Valerie Glatzel, Dirk Tomsitz, Michaela Maurer, Simone Schneider, Stefan Brunner, Nadia Harbeck, Lucie Heinzerling

**Affiliations:** ^1^ Department of Dermatology and Allergology LMU University Hospital Munich Germany; ^2^ Breast Center Department of Obstetrics and Gynecology LMU University Hospital Munich Germany; ^3^ Department of Internal Medicine I – Cardiology LMU University Hospital Munich Germany

**Keywords:** Hidradenocarcinoma, sweat gland, CDK4/6‐inhibitors, abemaciclib, letrozole, myocarditis

Dear Editors,

Hidradenocarcinoma is a very rare malignant tumor of the sweat gland,^1^ presenting as an erythematous or skin‐colored nodule, mostly on the scalp and neck. Only a few case reports exist with no approved treatment options. CDK4/6 inhibitors, such as abemaciclib, ribociclib, and palbociclib, are effective in hormone receptor‐positive, human epidermal growth factor receptor 2 (HER2)‐negative metastatic breast cancer.

This case report describes the effective therapy of a 75‐year‐old female patient with metastatic hidradenocarcinoma with a combination of CDK4/6 and aromatase inhibitors (Figure 1). She presented with extensive tumor of the capillitium and a cervical lymph node metastasis. Since hidradenocarcinomas have a specific expression of tumor markers, immunohistochemistry was performed. The tumor was positive for estrogen and progesterone receptor and negative for HER2; CK7 and GATA3 were positive, with no expression of CK5, CK6 and PD‐L1 (Figure 2). Pathologically, breast cancer metastasis could not be excluded but clinical assessment and a mammogram excluded breast carcinoma. CT scans confirmed the cervical lymph node metastasis with no signs for distant metastases.

**FIGURE 1:**
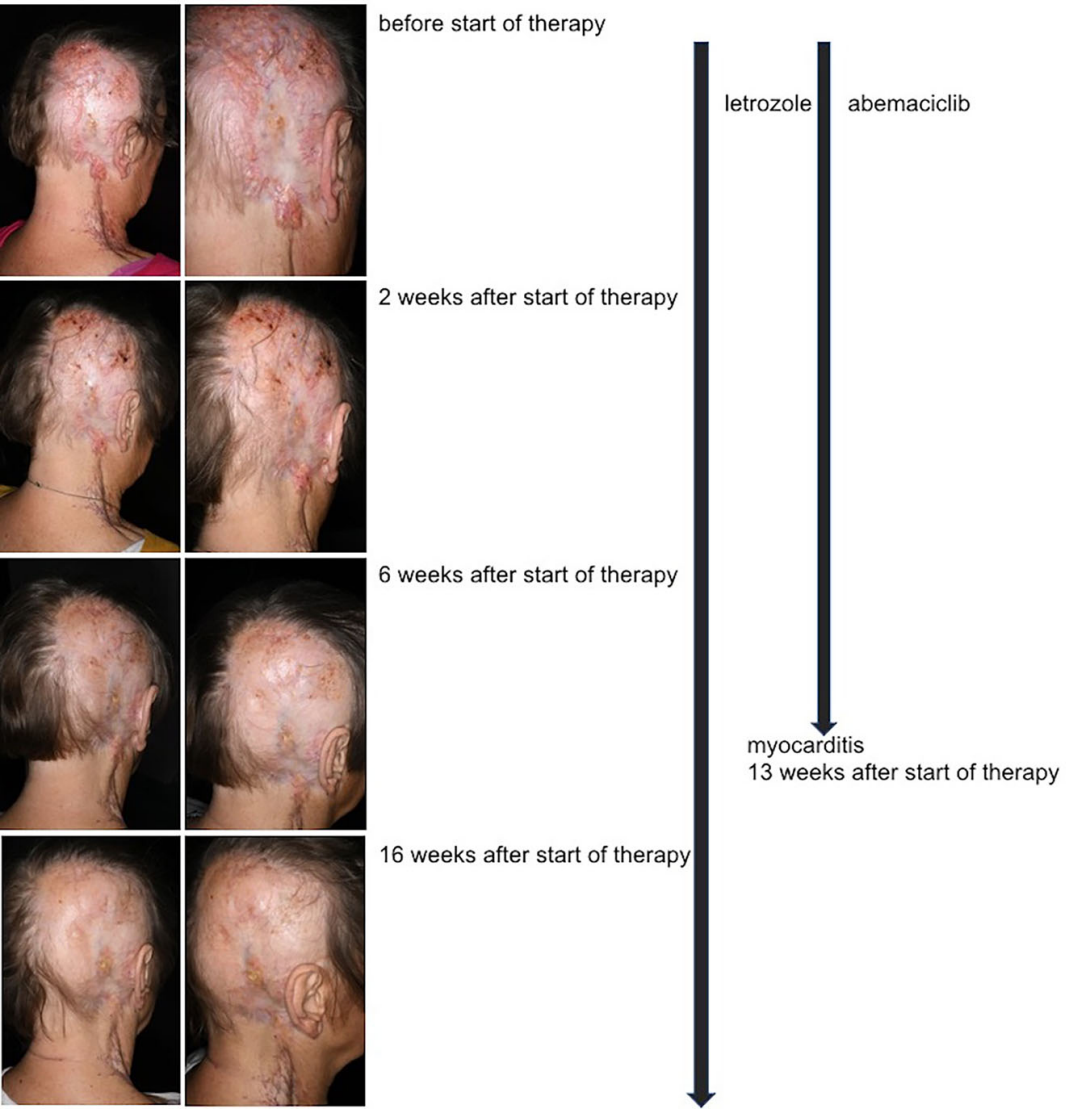
Clinical course of response of the hidradenocarcinoma to treatment with CDK4/6 inhibitor abemaciclib and aromatase inhibitor letrozole.

**FIGURE 2:**
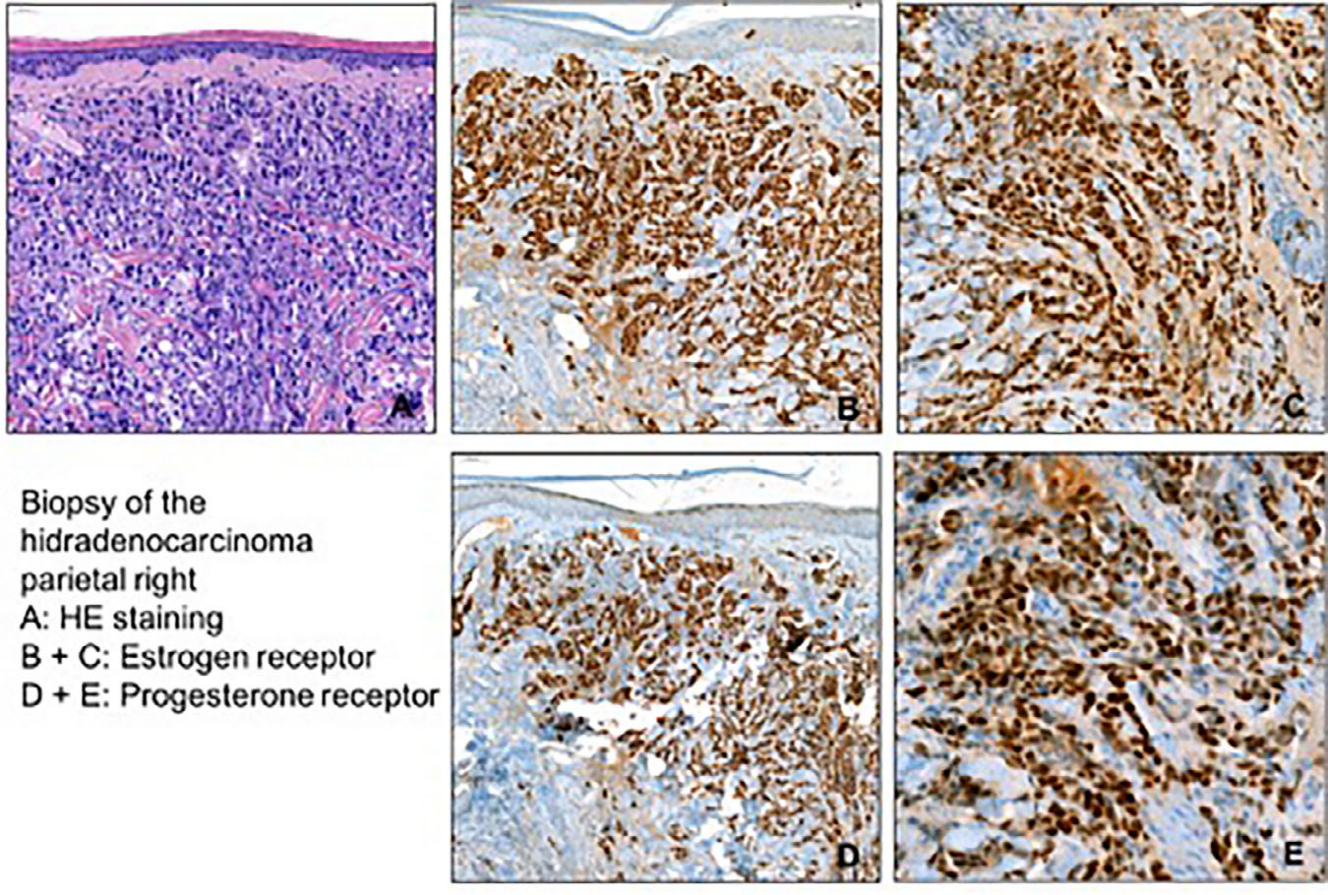
Histology of a ductal adenocarcinoma of the scalp, identified as hidradenocarcinoma, including positive staining of estrogen and progesterone receptors. CK7 and GATA 3 were positive, but the tumor was negative for CK5, CK6, and PD‐L1 expression.

In total, five excisions and resection of retroauricular subcutaneous metastases and cervical lymph node metastases of level V had been performed previously, followed by radiotherapy (50 Gy, 60 Gy, and 64 Gy). Tumor and cervical metastases recurred and were again surgically removed, including neck dissection.

Next‐generation sequencing using the “TruSight™ Oncology 500 molecular assay on the Illumina platform” revealed no targetable mutations, gene fusions, or amplifications. Tumor mutational burden was 2.4 mutations per Mb and the microsatellite status of the tumor was stable. Due to high expression of hormone receptors for estrogen and progesterone, therapy with CDK4/6‐ and aromatase inhibitor (letrozole) was recommended in analogy to the treatment of hormone receptor‐positive breast cancer. After preliminary examination with electrocardiography and echocardiography, CDK4/6 inhibitor abemaciclib 150 mg 1‐0‐1 in combination with letrozole 2 mg 1‐0‐0 was started.

A significant clinical response was observed after only 2 weeks (Figure 1). Regular laboratory controls showed a reduced glomerular filtration rate and increased creatinine. Frequencies of leukocytes and erythrocytes were low with 3.19 G/l and 3.30 T/l. Diarrhea at therapy initiation was well controlled with loperamide. After 13 weeks while profound tumor response was observed the patient reported stabbing chest pain. Cardiological work‐up in the emergency department revealed probably abemaciclib‐induced myocarditis with troponinemia and a moderate reduction in left ventricular ejection fraction. Consequently, the CDK4/6 inhibitor was discontinued. Since myocarditis has not been described for palbociclib, a class switch would have been possible. However, due to the good tumor response, therapy was continued with letrozole alone. The patient is in clinical remission with a follow‐up of 40 weeks.

Sweat gland tumors are hard to diagnose with different subtypes, partly overlapping features, and no established systemic therapy.^2^ Primary treatment consists of local excision, postoperative radiotherapy, and/or chemotherapy.^1^, ^2^, ^3^ However, tumors often recur and metastasize. Anti‐androgen therapy of an androgen receptor positive sweat gland tumor,^3^ and anti‐estrogen therapy for an estrogen receptor positive sweat gland tumor were successful in single cases.^4^

In our patient the CDK4/6 inhibitor was initiated in combination with the anti‐estrogen therapy letrozole, in analogy to the treatment of advanced breast cancer. CDK4/6 inhibitors interfere with cell growth by preventing phosphorylation of the retinoblastoma protein and inhibiting the transition from the G1 phase to the S phase.

Leukocytopenia, thrombocytopenia or anemia may occur, and creatinine has to be monitored. Thromboembolism has been described.^5^, ^6^ Although not part of the prescribing information or described in the literature, myocarditis has been reported in the manufacturer's safety database for abemaciclib. Thus, this is the first publication of abemaciclib‐induced myocarditis.

Hidradenocarcinoma is an extremely rare tumor with no standard treatment, especially in metastatic or recurrent disease. This case shows successful treatment with a CDK4/6 inhibitor in combination with an aromatase inhibitor.

## CONFLICT OF INTEREST STATEMENT

None.
